# Effect of Lactoferrin on Clinical Outcomes of Hospitalized Patients with COVID-19: The LAC Randomized Clinical Trial

**DOI:** 10.3390/nu15051285

**Published:** 2023-03-04

**Authors:** Erica Matino, Elena Tavella, Manuela Rizzi, Gian Carlo Avanzi, Danila Azzolina, Antonio Battaglia, Paolo Becco, Mattia Bellan, Giovanni Bertinieri, Massimo Bertoletti, Giuseppe Francesco Casciaro, Luigi Mario Castello, Umberto Colageo, Donato Colangelo, Davide Comolli, Martina Costanzo, Alessandro Croce, Davide D’Onghia, Francesco Della Corte, Luigi De Mitri, Valentina Dodaro, Filippo Givone, Alessia Gravina, Luca Grillenzoni, Graziano Gusmaroli, Raffaella Landi, Anna Lingua, Roberto Manzoni, Vito Marinoni, Bianca Masturzo, Rosalba Minisini, Marina Morello, Anna Nelva, Elena Ortone, Rita Paolella, Giuseppe Patti, Anita Pedrinelli, Mario Pirisi, Lidia Ravizzi, Eleonora Rizzi, Daniele Sola, Mariolina Sola, Nadir Tonello, Stelvio Tonello, Gigliola Topazzo, Aldo Tua, Piera Valenti, Rosanna Vaschetto, Veronica Vassia, Erika Zecca, Nicoletta Zublena, Paolo Manzoni, Pier Paolo Sainaghi

**Affiliations:** 1 Department of Translational Medicine, Università del Piemonte Orientale (UPO), 28100 Novara, Italy; 2 Department of Internal Medicine and COVID-19 Unit, Azienda Ospedaliero-Universitaria (AOU) “Maggiore della Carità”, 28100 Novara, Italy; 3 Division of Emergency Medicine and COVID-19 Sub-Intensive Unit, Azienda Ospedaliero-Universitaria (AOU) “Maggiore della Carità”, 28100 Novara, Italy; 4 Department of Maternal-Infant Medicine, Ospedale degli Infermi, 13875 Ponderano, Italy; 5 Internal Medicine, Department of Medical Sciences, Azienda Ospedaliero-Universitaria (AOU) Città della Salute e della Scienza, University of Turin School of Medicine, 10126 Turin, Italy; 6 Department of Health Sciences, Università del Piemonte Orientale (UPO), 28100 Novara, Italy; 7 Division of Dermatology, Ospedale degli Infermi, 13875 Ponderano, Italy; 8 Division of Oncology, Ospedale degli Infermi, 13875 Ponderano, Italy; 9 CAAD, Center for Autoimmune and Allergic Diseases, Università del Piemonte Orientale (UPO), 28100 Novara, Italy; 10 Division of Internal Medicine, Ospedale degli Infermi, 13875 Ponderano, Italy; 11 Division of Pneumology, Ospedale degli Infermi, 13875 Ponderano, Italy; 12 Division of Internal Medicine, Azienda Ospedaliera “SS. Antonio e Biagio e Cesare Arrigo”, 15121 Alessandria, Italy; 13 Intensive Care Unit, Ospedale degli Infermi, 13875 Ponderano, Italy; 14 Department of Anesthesia and Intensive Care Medicine, AOU “Maggiore della Carità”, 28100 Novara, Italy; 15 Division of Diabetology and Endocrinology, Ospedale degli Infermi, 13875 Ponderano, Italy; 16 Division of Emergency Medicine, Ospedale degli Infermi, 13875 Ponderano, Italy; 17 Division of Neurology, Ospedale degli Infermi, 13875 Ponderano, Italy; 18 Division of Infectious Disease, Ospedale degli Infermi, 13875 Ponderano, Italy; 19 Division of Geriatric Care, Ospedale degli Infermi, 13875 Ponderano, Italy; 20 Division of Obstetrics and Gynecology, Ospedale degli Infermi, 13875 Ponderano, Italy; 21 Medical Department, Division of Cardiology, AOU “Maggiore della Carità”, 28100 Novara, Italy; 22 Department of Public Health and Infectious Diseases, University of Rome, La Sapienza, 00185 Rome, Italy; 23 Division of Palliative Care, Ospedale degli Infermi, 13875 Ponderano, Italy

**Keywords:** COVID-19, lactoferrin, randomized, placebo-controlled, multicenter, double-blind clinical trial

## Abstract

As lactoferrin is a nutritional supplement with proven antiviral and immunomodulatory abilities, it may be used to improve the clinical course of COVID-19. The clinical efficacy and safety of bovine lactoferrin were evaluated in the LAC randomized double-blind placebo-controlled trial. A total of 218 hospitalized adult patients with moderate-to-severe COVID-19 were randomized to receive 800 mg/die oral bovine lactoferrin (n = 113) or placebo (n = 105), both given in combination with standard COVID-19 therapy. No differences in lactoferrin vs. placebo were observed in the primary outcomes: the proportion of death or intensive care unit admission (risk ratio of 1.06 (95% CI 0.63–1.79)) or proportion of discharge or National Early Warning Score 2 (NEWS2) ≤ 2 within 14 days from enrollment (RR of 0.85 (95% CI 0.70–1.04)). Lactoferrin showed an excellent safety and tolerability profile. Even though bovine lactoferrin is safe and tolerable, our results do not support its use in hospitalized patients with moderate-to-severe COVID-19.

## 1. Introduction

Severe acute respiratory syndrome coronavirus (SARS-CoV-2) is a positive, single-stranded β-coronavirus, showing high genetic similarities to both SARS-CoV and Middle East respiratory syndrome coronavirus (MERS-CoV) [1,2]. Its viral entry into host cells depends on the interaction of the viral spike protein with cellular receptors (i.e., angiotensin-converting enzyme 2, ACE2) and co-receptors (i.e., heparan sulphate proteoglycans, HSPGs) [3,4]. The clinical spectrum of its associated coronavirus disease 19 (COVID-19) is wide, ranging from a mild upper respiratory tract infection to severe interstitial pneumonia, with respiratory and even multi-organ failure [3,5,6]. 

Although the vaccination campaign and the implementation of targeted treatments early in the disease course have lowered the risk of developing severe COVID-19, the current in-hospital mortality of unvaccinated subjects remains quite high [7,8]. To date, there are few specific and effective treatments for patients with severe COVID-19 requiring hospitalization. For these patients, the current guidelines recommend steroids and anti-cytokine treatments to mitigate the clinical deterioration associated with systemic hyperinflammation [9,10,11].

In this scenario, several drugs and natural products have been proposed as candidate treatments for COVID-19, owing to their presumed antiviral efficacy or immunomodulatory effects [12,13,14]. One of these is lactoferrin, an iron-binding glycoprotein of the transferrin family, with a high homology across mammalian species. In particular, its concentration is at its highest in colostrum and milk, where it protects newborns from infections [15,16,17]. 

Several studies have shown how bovine lactoferrin can interfere with SARS-CoV and SARS-CoV-2 infections in vitro by either enhancing natural killer (NK) cell and neutrophil activities, boosting interferon-mediated immune responses, or blocking viral internalization via binding to HSPGs [13,18,19,20,21,22,23]. Furthermore, in silico studies have suggested a possible mechanism of action based on lactoferrin being able to directly bind to SARS-CoV-2 spike glycoproteins [21] and compete for ACE2 binding [24].

Bovine lactoferrin is commercially available as a generally recognized as safe (GRAS) nutritional supplement, with a high homology to the human protein and very similar biological activities [17,25]. Indeed, it is well-tolerated in different clinical contexts, such as the prevention and management of necrotizing enterocolitis and neonatal sepsis, especially in premature, low-birth-weight newborns [26,27,28,29,30,31,32,33,34,35,36,37].

In light of the above properties, lactoferrin has been tested in a few non-randomized pilot studies in patients with COVID-19 with controversial results [25,38,39]. To further evaluate the role of lactoferrin in COVID-19, we designed a randomized, double-blind, placebo-controlled, multicenter clinical trial to investigate the efficacy of a daily oral dose of bovine lactoferrin in combination with the standard of care in improving clinical recovery and reducing adverse outcomes in hospitalized patients affected by moderate-to-severe COVID-19. 

## 2. Materials and Methods

### 2.1. Ethics and Study Design

The lactoferrin for treatment of acute COVID-19 in hospitalized patients trial (LAC trial) is a non-profit, randomized, double-blind, multicenter, placebo-controlled, parallel-arm clinical trial (ClincalTrials.gov registration: NCT04847791). This study was approved by the Local Ethics Committee (Comitato Etico Interaziendale Novara, Identifier: CE 6/21) and conducted in accordance with the Declaration of Helsinki. The study took place from January to May 2021, during the third wave of the COVID-19 pandemic in Italy. 

**Figure 1:**
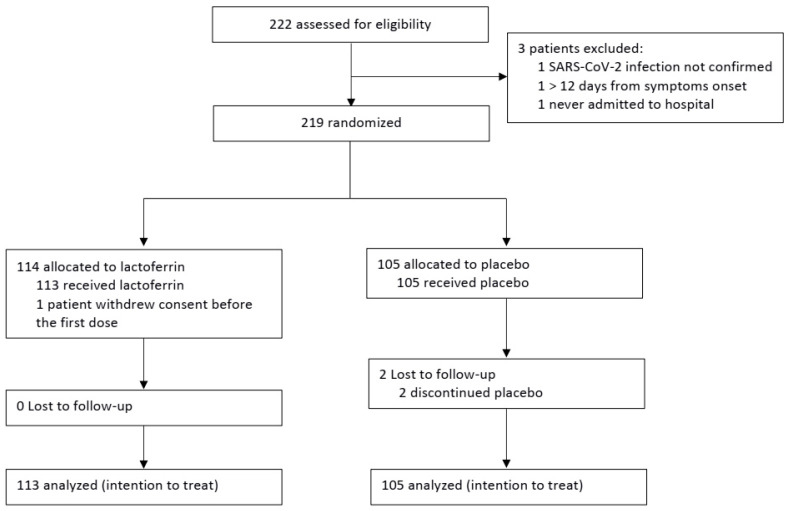
Flow diagram. The flow diagram shows the number of subjects assessed for eligibility, randomized, randomized and receiving intervention, randomized and withdrawing consent before treatment initiation, and included in the primary analysis. Patients who were eligible but not randomized, who were not randomly allocated, who were lost to follow-up, and who discontinued the intervention are also reported.

The aim of this study was to evaluate the efficacy of an oral daily dose of bovine lactoferrin vs. placebo in limiting the progression and severity of COVID-19 infection and/or favoring clinical recovery in hospitalized patients. Lactoferrin therapy was administered in combination with standard COVID-19 therapy. This report follows the Consolidated Standards of Reporting Trials (CONSORT) reporting guidelines (Figure 1). The trial protocol is included in the Supporting Information Section (S1 File) [3,17,18,39,43,44,61,62,63,64,65,66,67,68,69,70,71,72,73,74,75,76,77,78,79,80,81,82,83,84].

### 2.2. Population

Patients were recruited in two Italian hospitals: “Ospedale degli Infermi” (Ponderano, Biella) and “AOU Maggiore della Carità” (Novara). 

The inclusion criteria were as follows: hospitalization in a non-intensive care unit (non-ICU) in COVID-19-dedicated wards, with SARS-CoV-2 infection confirmed through either reverse-transcription polymerase chain reaction (RT-PCR) or a third-generation antigenic test (according to local guidelines) from nasopharyngeal swab samples; age ≥ 18 years; and onset of COVID-19 symptoms within 12 days before hospitalization.

The exclusion criteria were as follows: refusal to give informed consent; the need for immediate ICU admittance; advanced cancer/malignancy history; known allergies or intolerances to lactoferrin; being already exposed to lactoferrin when hospital admission occurred; end-stage renal disease (stage V, GFR < 15 mL/min); critical clinical conditions suggestive of imminent death; and inability to tolerate and/or clinical conditions that might contraindicate oral treatments.

### 2.3. Randomization

Patients admitted to the COVID-19 wards were screened for LAC trial eligibility by a member of the clinical staff. They were asked to sign and date a specific informed consent form, and then they were randomized 1:1 to the bovine lactoferrin or placebo arm. Allocation to one of the treatment groups was performed using a restricted randomization procedure based on the permuted block randomization scheme with a block size of 4 to ensure a balanced allocation for each participating center [40,41]. Each block consisted of a specific number of allocation treatments, randomly sorted. The hospital pharmacy of each center was not involved in patient recruitment, but it prepared sequentially numbered opaque-sealed envelopes containing the assignation code (Group A or Group B), equally split between the two study arms. The sealed envelopes were sequentially opened at the time of randomization, after having obtained informed consent. The hospital pharmacy staff was responsible for providing both Mosiac 200 mg (bovine lactoferrin) and placebo capsules in white bottles that were not distinguishable to the investigators, with the only exception of a Group A or Group B label. The patients and investigators were blinded to the association of the treatment code to lactoferrin or placebo. Blindness was also preserved by physically separating the pharmacy staff preparing the randomization list from the investigators. Moreover, the principal investigators were blinded to the treatment protocol. The screening and randomization processes were performed within 24 h from patient admission, while the standard of care therapy was started immediately regardless of study procedures.

### 2.4. Intervention

The patients allocated to the treatment group received a daily dose of 800 mg of bovine lactoferrin. Specifically, the patients were given 2 capsules of Mosiac (Pharmaguida Srl, Rome, Italy), 200 mg every 12 h before meals for 30 days. The patients allocated to the control group received the placebo, consisting of inert components (cornstarch powder) administered as capsules identical to the lactoferrin ones, with the same posology. Each capsule was evaluated for lactoferrin content corresponding to about 200 mg of lactoferrin. The purity of the lactoferrin, checked using SDS-PAGE and silver nitrate staining, was 98.5%. The concentration of the lactoferrin was assessed using UV spectroscopy on the basis of an extinction coefficient of 15.1 (280 nm, 1% solution). The lactoferrin iron saturation was about 12%, as detected using optical spectroscopy at 468 nm on the basis of an extinction coefficient of 0.54 (100% iron saturation, 1% solution). Both groups of patients received the same product batch, produced ex novo ad hoc and specifically prepared for the study. All these quality assessments were run prior to the beginning of the study by third-party entities at La Sapienza University in Rome, who remained blinded to all study procedures. Both oral lactoferrin and placebo were kindly supplied by Pharmaguida Srl. The interventions were administered in addition to the standard of care in clinical practice for patients with COVID-19. Pharmacological therapies were prescribed following the most updated guidelines and the best clinical practice at the time of study conduction, according to each patients’ individual condition. 

### 2.5. Safety

Bovine lactoferrin is a nutraceutical supplement with the status of GRAS officially granted by the US Food and Drug Administration (FDA). No adverse drug reactions were expected following its administration. Nonetheless, we performed a systematic detection of adverse events and severe adverse effects. The need for treatment withdrawal, along with laboratory findings during the time of the study, was reported in the medical records.

### 2.6. Clinical and Laboratory Monitoring

For each enrolled patient, clinical information and laboratory findings, easily obtainable from medical records, were collected at different time points during hospitalization starting from the time of admission (baseline, t0) until discharge (or for a maximum of 28 days) or study exit (death or ICU admission), whichever occurred first. The type of data and the frequency of collection are summarized in Table 1.

### 2.7. Data Management

The data of interest were recorded in a web-based database created ad hoc for the study on the REDCap platform [42], accessible only to study investigators through user-sensitive passwords. Data were pseudo-anonymized for recording, and a list containing the pairing between the trial identification code and personal data was stored in a secured place to allow for unmasking in the case of emergency situations. 

### 2.8. Definition of Endpoints

The primary endpoints were evaluated in the intention-to-treat (ITT) population to assess the effect of oral bovine lactoferrin in modifying at least one of the following: (1) a decrease in the proportion of a composite event rate consisting of one of two events, whichever occurred first: hospitalization in ICU (due to any cause) or in-hospital death, and (2) an increase in the proportion of a composite event rate consisting of one of two events, whichever occurred first: discharge from hospital within 14 days from enrollment or a National Early Warning Score 2 (NEWS2) ≤ 2 for at least 24 h within 14 days from enrollment. 

The secondary endpoints were defined as differences in outcomes between the treatment and the placebo groups as follows: (i) variations, either improvements or worsening, in the NEWS2 score from baseline values to those measured at 7, 14, and 21 days; (ii) the need for oxygen supplementation and, if so, its duration; (iii) the need for non-invasive ventilation (NIV), including a high-flow nasal cannula (HFNC) and a properly defined NIV, encompassing either continuous positive airway pressure (CPAP) or bi-level positive airway pressure (BiPAP) using a non-invasive interface; (iv) the need for invasive mechanical ventilation; (v) variations in in-hospital mortality rates at 14 and 28 days from enrollment; (vi) variations in C-reactive protein (CRP), interleukin 6 (IL-6), and ferritin plasma levels during hospitalization; and (vii) adverse events related to the treatments. The primary endpoints were also evaluated in two different age subgroups (<65 years vs. ≥65 years) and in relation to gender.

### 2.9. Sample Size

The trial was powered for the primary endpoint. Pre-trial data from the two recruiting centers estimated a 25% ICU admission rate, a 30% non-invasive ventilation requirement rate, a 15% in-hospital mortality rate, and an average hospital stay of 16 days. The sample size was estimated considering a two-sided *t*-test for two independent samples according to two possible scenarios: (1) 80 patients/arm with an alpha level of 0.05, an overall power of 0.8, and a Cohen effect of 0.44 corresponding to a 14 day discharge rate of 60% for controls and 80% for treated patients, and (2) 97 patients/arm with an alpha level of 0.025 corrected according to the Bonferroni method, an overall power of 0.8, and a Cohen effect of 0.44. Sample size calculations were performed using R 3.6.1 software [43] and the pwr package [44].

### 2.10. Statistical Analysis

Continuous variables are expressed in terms of median and interquartile range (IQR), while categorical variables are presented as percentages (absolute numbers). 

The primary endpoints were evaluated on an ITT population basis by performing a bilateral *t*-test to evaluate the differences between proportions and to calculate the relative risk (RR) with a 95% confidence interval (95% CI).

The secondary endpoints were evaluated by performing a bilateral *t*-test to evaluate the differences between proportions for binary endpoints (Pearson χ2 test) and by carrying out the Mann–Whitney U test to evaluate differences between median values for continuous endpoints. The threshold significance was set at 0.05. Statistical tests were performed with either the software package Statistica for Windows, release 12 (TIBCO Software Inc., Palo Alto, CA, USA), or MedCalc^®^ Statistical software, version 20.014 (MedCalc Software Ltd., Ostend, Belgium).

## 3. Results

Out of 222 patients assessed for eligibility (Figure 1), 3 patients were excluded because they did not meet the inclusion criteria. Thus, a total of 219 patients underwent randomization after providing informed consent. Of these, 114 were allocated to the lactoferrin group and 105 to the placebo one. One patient withdrew consent before treatment initiation and was thus excluded from the final analysis. Consequently, 218 patients were included in the ITT analysis (113 in the lactoferrin arm and 105 in the placebo arm) (Figure 1). 

Table 2 reports the baseline demographic, clinical, and laboratory features of the patients included in the ITT analysis allocated to the treatment groups, while the baseline data of the general enrolled population are reported in Table 3. As evident, the demographic, clinical, and laboratory characteristics were similar between groups.

### 3.1. Primary Outcomes

An almost equal number of patients with COVID-19 in each group were transferred to the ICU or died during hospitalization—24/113 (21.2%) lactoferrin-treated patients vs. 21/105 (20.0%) patients in the placebo group; proportion difference = 1.20% (95% CI: −9.63–11.85), *p* = 0.8272; RR: 1.06 (95% CI: 0.63–1.79)—clearly indicating that lactoferrin is not effective in modifying the predefined negative composite event rate. Similarly, lactoferrin treatment did not increase the probability of reaching the predefined composite positive outcome, as 67/113 (59.3%) lactoferrin-treated patients vs. 73/105 (69.5%) patients in the placebo group reached NEWS2 ≤ 2 or were discharged from hospital within 14 days from randomization—absolute proportion difference 10.20% (95% CI: −2.52–22.40), *p* = 0.1173; RR: 0.85 (95% CI: 0.70–1.04).

### 3.2. Secondary Outcomes

We did not observe any significant variations in the NEWS2 scores of the lactoferrin vs. placebo groups recorded at 7, 14, and 21 days with respect to baseline. Likewise, we did not detect any lactoferrin effects on the need for oxygen supplementation and its duration, non-invasive or mechanically assisted ventilation, or in-hospital mortality at 14 and 28 days from admission (Table 4).

**Figure 2:**
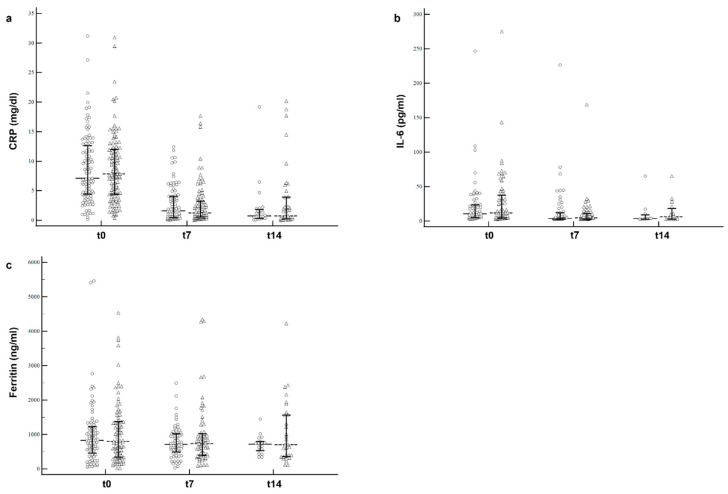
Changes in inflammatory markers between lactoferrin and placebo arms. CRP (**a**), IL-6 (**b**), and ferritin (**c**) plasma levels measured at different time points (t0, t7, t14). Circles represent the placebo arm, while triangles represent the lactoferrin one. Data are reported as median values (dashed lines) and interquartile range (bold lines).

With regard to the laboratory parameters, we did not record a significant difference in CRP, IL-6, or ferritin plasma concentrations measured at 7 days and 14 days between treatment groups (Figure 2).

A subgroup analysis of subjects 65 years or older vs. those younger than 65 years and of females vs. males failed to reveal any effects of lactoferrin on primary outcomes (Table 5).

Lastly, there were no significant differences in the proportion of adverse events between the lactoferrin and placebo arms (Table 6), indicating that lactoferrin is well-tolerated and has a safe profile.

## 4. Discussion

During the first phases of the pandemic, the use of over-the-counter nutritional supplements to treat COVID-19 grew in popularity in Italy. In particular, bovine lactoferrin soon became one of the most sought-after supplements despite only a few pilot studies suggesting a potential beneficial effect on the clinical course of COVID-19, regardless of pharmacological formulation and disease severity [25,38,39].

Thus, the aim of our study was to shed light on this issue by using a methodologically sound approach. For this purpose, we conducted a prospective, placebo-controlled, multicenter, double-blind clinical trial on a cohort of patients with COVID-19 hospitalized in non-ICU wards. Our results show that a daily 800 mg dose of bovine lactoferrin vs. placebo administered in combination with standard COVID-19 therapy could neither mitigate disease evolution (i.e., the prevention of death or ICU transfer) nor support clinical recovery. Furthermore, we did not observe any significant lactoferrin effects in modifying clinical variables, such as NEWS2 score and the need for oxygen supplementation, or pro-inflammatory biomarkers (e.g., CRP, IL-6, and ferritin). Similar results were also obtained in the elderly patient subgroup treated in the late stage (6-day median) of COVID-19 or after gender stratification.

Altogether, our findings do not support the use of bovine lactoferrin in hospitalized patients with moderate-to-severe COVID-19. As our study population consists of a homogeneous cohort of patients, in terms of both clinical features and therapeutic regimen, equally distributed between the two study arms, it provides a simpler framework with which to interpret the efficacy of lactoferrin as an adjuvant in the late phase of COVID-19 treatment. Notably, a recent Egyptian randomized, prospective, interventional study in a few hospitalized patients with mild-to-moderate COVID-19 also obtained similar results [45], even though the authors employed lower doses of lactoferrin and a shorter treatment schedule than other non-randomized trials [25,38].

The lack of clinical efficacy of lactoferrin may be due to several factors, with one being a suboptimal bioavailability of bovine lactoferrin. However, despite the paucity of pharmacokinetic studies on its absorption in humans, lactoferrin is known to be well absorbed by the intestine in both mice and pigs [46,47]. Furthermore, it is well-established that the human intestine expresses the lactoferrin receptor [48,49] and that such receptor is able to internalize both human [50,51] and, albeit with a lower efficiency, bovine lactoferrin [52]. Thus, we feel that the high dose of 800 mg used in this trial should have overcome bioavailability issues.

Another possible reason for the poor efficacy may be ascribed to the timing of lactoferrin administration. Indeed, in the three pilot studies where a clinical course improvement was observed [25,38,39], bovine lactoferrin was given at an earlier stage of disease, just following SARS-CoV-2 detection. However, in our study, lactoferrin was administered on the day of hospitalization, at a median of 6 days from symptom onset, thus in patients with a more advanced disease, when the dysregulated immune response starts to become independent from viral replication. This would be consistent with previous studies on remdesivir and molnupiravir, two drugs that target SARS-CoV-2, which have little effect in improving clinical evolution when administered in the late disease phase, while they display a strong efficacy against COVID-19 progression once given in the early phase of the disease [53,54,55]. Thus, our results do not rule out a potential antiviral effect of lactoferrin if given at early stages of disease.

Another important observation of our study is the lack of modulation of pro-inflammatory markers by lactoferrin despite its well-established role as an immunomodulator [19,56]. This may be explained by the fact that we used lactoferrin as an adjuvant to standard therapy, which consists of high doses of corticosteroids and heparin. Indeed, the anti-inflammatory activity of corticosteroids [57,58] could have, at least in part, masked any potential immunomodulatory effects of lactoferrin. Furthermore, the presence of heparin, which was administered in a prophylactic or therapeutic dose to all patients, may have similarly affected the antiviral activity of lactoferrin [19,59], based on a heparin-dependent reduction in lactoferrin antiviral activity [22], probably due to heparin competition for HSPG binding [60].

Finally, it is worth pointing out that, in our study, we did not record any relevant adverse events related to lactoferrin treatment. This highlights the good safety and tolerability profile of this compound, thus supporting the design of future clinical trials to assess the adjuvant role of lactoferrin in the early phases of the disease.

Even though our trial protocol was designed to minimize the potential risk of biases, as it was conducted in clinical practice settings during the third pandemic wave, we cannot rule out that some slight differences in the treatment of individual patients may have occurred due to several different coexisting diseases. However, it is unlikely that these variables would have biased our results.

## 5. Conclusions

During the COVID-19 pandemic, lactoferrin was widely proposed as an antiviral agent and a booster of the immune response. This randomized, placebo-controlled, multicenter clinical trial did not show any significant effect of lactoferrin on modifying the clinical evolution and/or laboratory markers of inflammation when used as an add-on treatment in adult patients hospitalized with moderate-to-severe COVID-19. Thus, our data do not support the use of lactoferrin in patients with COVID-19 during hospitalization. Further studies are, however, warranted to explore the possibility that lactoferrin may be useful in earlier phases of COVID-19, when a specific antiviral activity might be more relevant. 

## Figures and Tables

**Table 1:** Data collection frequency for the relevant parameters.

At Admission	Daily during Hospitalization	Weekly during Hospitalization	At Discharge
- Informed consent - Fulfillment of inclusion criteria - Randomization - Medical history - Ongoing pharmacological therapies - NEWS2 - Need for oxygen supplementation - Arterial blood gas analysis - Nasal swab analysis - Routine hematological analysis	- NEWS2 - Vital signs - P/F ratio - Oxygen supplementation - Adverse events	- Arterial blood gas analysis - Routine hematological analysis	- NEWS2 - Adverse events - Oxygen supplementation - Arterial blood gas analysis - Routine hematological analysis

**Table 2:** Demographic and baseline characteristics of the studied population. Data are expressed as number of patients or median (interquartile range, IQR) when appropriate. * refers to data obtained with oxygen supplementation.

	Lactoferrin (n = 113)	Placebo (n = 105)	
**Female/Male**	44/69	33/72	χ^2^ 1.3435, *p* = 0.2464
**Age, median (IQR), years**	66 (56–73)	65 (57–73)	Z = 0.4427, *p* = 0.6580
**Symptoms**			
Productive cough	12/101	11/94	χ^2^ 0.0012, *p* = 0.9726
Non-productive cough	41/72	43/62	χ^2^ 0.5010, *p* = 0.4791
Dyspnea	79/34	71/34	χ^2^ 0.1333, *p* = 0.7151
Diarrhea	18/95	22/83	χ^2^ 0.9166, *p* = 0.3384
**COVID-19 related home treatment**			
Hydroxychloroquine	0/113	2/103	χ^2^ 2.1723, *p* = 0.1405
Azithromycin	28/85	35/70	χ^2^ 1.9384, *p* = 0.1638
Heparin	34/79	25/80	χ^2^ 1.0871, *p* = 0.2971
Steroids	53/60	45/60	χ^2^ 0.3580, *p* = 0.5494
**Number of medications**			χ^2^ 12.8569, *p* = 0.2318
None	30	26	
1	20	20	
2–4	38	32	
≥4	25	27	
**Comorbidities**			
BMI ≥ 30	38	27	χ^2^ 1.6212, *p* = 0.2029
Current or former smokers	19	22	χ^2^ 0.6290, *p* = 0.4279
Charlson Comorbidity Index, median (IQR)	2 (1–4)	3 (1–4)	Z = −0.0792, *p* = 0.9368
**Days from symptoms onset, median (IQR)**	6 (4–7)	7 (4–8)	Z = −1.7939, *p* = 0.0728
**Vital parameters**			
Temperature, median (IQR), °C	36.5 (36.1–37.1)	36.5 (36.1–36.9)	Z = 0.4530, *p* = 0.6505
Cardiac rate, median (IQR), beats/min	81 (75–91)	85 (73–96)	Z = −0.8456, *p* = 0.3978
Respiratory rate, median (IQR), breaths/min *	20 (18–24)	20 (18–25)	Z = 0.9209, *p* = 0.3571
spO_2_%, median (IQR) *	95 (93–97)	96 (93–97)	Z = −1.7675, *p* = 0.0771
Systolic pressure, median (IQR), mmHg	125 (116–140)	126 (117–142)	Z = −0.2227, *p* = 0.8238
Diastolic pressure, median (IQR), mmHg	75 (70–80)	75 (70–85)	Z = −0.5018, *p* = 0.6158
**NEWS2, median [IQR]**	5 (4–6)	5 (3–6)	Z = 0.0790, *p* = 0.9371
**Laboratory findings**			
Hemoglobin, median (IQR), g/dL	13.9 (12.7–14.9)	14.0 (12.3–15.0)	Z = 0.2630, *p* = 0.7925
Leukocytes, median (IQR), cells×10^3^/µL	7.4 (5.2–10.1)	6.6 (4.9–8.9)	Z = 1.1709, *p* = 0.2417
Neutrophils, median (IQR), cells×10^3^/µL	6.0 (4.3–8.7)	5.4 (3.9–7.6)	Z = 1.2130, *p* = 0.2251
Lymphocytes, median (IQR), cells×10^3^/µL	0.7 (0.6–1.0)	0.7 (0.5–1.0)	Z = 0.0807, *p* = 0.9357
Platelets, median (IQR), cells×10^3^/µL	200 (156–255)	210 (167–269)	Z = −0.8852, *p* = 0.3760
ALT, median (IQR), U/L	33 (24–54)	35 (25–52)	Z = −0.3495, *p* = 0.7267
AST, median (IQR), U/L	41 (31–57)	39 (28–54)	Z = 0.9099, *p* = 0.3629
Creatinine, median (IQR), mg/dL	0.8 (0.7–1.0)	0.8 (0.7–1.0)	Z = 0.0303, *p* = 0.9758
Erythrocyte sedimentation rate, median (IQR), mL/min	41 (31–55)	38 (24–49)	Z = 1.5780, *p* = 0.1146
CRP, median (IQR), mg/dL	7.8 (4.4–12.0)	7.1 (4.4–12.9)	Z = 0.1053, *p* = 0.9161
LDH, median (IQR), U/L	533 (345–830)	634 (459–821)	Z = −1.4739, *p* = 0.1405
Troponin I, median (IQR), ng/mL	6 (3–17)	8 (4–14)	Z = −0.4006, *p* = 0.6887
Ferritin, median (IQR), ng/mL	797 (343–1373)	827 (461–1229)	Z = 0.2026, *p* = 0.8395
D-dimer, median (IQR), µg/L	658 (438–1148)	800 (544–1390)	Z = −1.3029, *p* = 0.1926
IL-6, median (IQR), pg/mL	11.5 (3.1–28.2)	9.1 (4.2–22.6)	Z = 0.0970, *p* = 0.9227
**Arterial blood gas analysis** *			
pH, median (IQR)	7.5 (7.4–7.5)	7.5 (7.4–7.5)	Z = −0.5288, *p* = 0.5969
pO_2_, median (IQR), mm Hg	71 (61–80)	66 (60–76)	Z = 1.3906, *p* = 0.1643
pCO_2_, median (IQR), mm Hg	37 (34–39)	36 (33–39)	Z = 0.7675, *p* = 0.4434
P/F, median (IQR)	155 (124–209)	151 (124–243)	Z = −0.2884, *p* = 0.7884

Abbreviations: NEWS2 = National Early Warning Score 2, ALT = alanine transaminase, AST = aspartate aminotransferase, CRP = C-reactive protein, LDH = lactic dehydrogenase.

**Table 3:** Demographic and baseline characteristics of the studied population. Data are expressed as number of patients or median (interquartile range, IQR) when appropriate. * refers to data obtained with oxygen supplementation.

Demographics, Parameters, and Clinical Scores	Values
**Female/Male**	77/141
**Age, median (IQR), years**	65.5 (56.4–73.4)
**Symptoms**	
Productive cough	23/195
Non-productive cough	84/134
Dyspnea	150/68
Diarrhea	40/178
**COVID-19 related home treatment**	
Hydroxychloroquine	2/216
Azithromycin	63/155
Heparin	59/159
Steroids	98/120
**Number of medications**	
None	56
1	40
2–4	70
≥4	52
**Comorbidities**	
BMI ≥ 30	65
Current or former smokers	41
Charlson Comorbidity Index, median (IQR)	3 (1–4)
**Days from symptoms onset**	6 (4–8)
**Vital parameters**	
Temperature, median (IQR), °C	36.5 (36.1–37.0)
Cardiac rate, median (IQR), beats/min	82 (74–94)
Respiratory rate, median (IQR), breaths/min *	20 (18–24)
spO_2_%, median (IQR) *	95 (93–97)
Systolic pressure, median (IQR), mmHg	125 (116–140)
Diastolic pressure, median (IQR), mmHg	75 (70–82)
**NEWS2**, median (IQR)	5 (4–6)
**Laboratory findings**	
Hemoglobin, median (IQR), g/dL	13.9 (12.5–15)
Leukocytes, median (IQR), cells×10^3^/µL	7.0 (5.1–9.5)
Neutrophils, median (IQR), cells×10^3^/µL	5.7 (4.2–8.3)
Lymphocytes, median (IQR), cells×10^3^/µL	0.7 (0.6–1.0)
Platelets, median (IQR), cells×10^3^/µL	207 (161–260)
ALT, median (IQR), U/L	34 (24–52)
AST, median (IQR), U/L	39 (30–56)
Creatinine, median (IQR), mg/dL	0.8 (0.7–1.0)
Erythrocyte sedimentation rate, median (IQR), mL/min	40 (25–53)
CRP, median (IQR), mg/dL	7.8 (4.4–12.0)
LDH, median (IQR), U/L	585 (379–829)
Troponin I, median (IQR), ng/mL	8 (3–15)
Ferritin, median (IQR), ng/mL	820.5 (394–1341)
D-dimer, median (IQR), µg/L	708 (496–1331)
IL-6, median (IQR), pg/mL	11.3 (5.0–31.6)
Arterial blood gas analysis *	
pH, median (IQR)	7.5 (7.4–7.5)
pO_2_, median (IQR), mm Hg	68.2 (60.0–79.5)
pCO_2_, median (IQR), mm Hg	36.6 (33.1–39.0)
P/F, median (IQR)	154 (124–222)

Abbreviations: NEWS2 = National Early Warning Score 2, ALT = alanine transaminase, AST = aspartate aminotransferase, CRP = C-reactive protein, LDH = lactic dehydrogenase.

**Table 4:** Secondary outcomes. Data are expressed as number of patients or median (interquartile range, IQR) when appropriate.

	Lactoferrin (n = 113)	Placebo (n = 105)	
**Variation of NEWS2 from baseline** **at 7 days, median (IQR)** **at 14 days, median (IQR)** **at 21 days, median (IQR)**	−2 (−3–0) (n = 85) −2 (−3–0) (n = 41) −1 (−4–1) (n = 19)	−2 (−4–1) (n = 86) −2 (−3–0) (n = 26) −2 (−4–1) (n = 11)	Z = 0.4337, *p* = 0.6645 Z = 0.5793, *p* = 0.5624 Z = −0.3055, *p* = 0.7600
**Days of oxygen supplementation, median (IQR)**	11(7–14)	12 (8–19)	Z = −1.314, *p* = 0.1888
**Patients needing** **any oxygen supplementation** **HFNC or NIV** **mechanical ventilation**	104 88 14	99 73 9	RR = 0.98, 95% CI 0.91–1.05 RR = 1.12, 95% CI 0.95–1.131 RR = 1.45, 95% CI 0.65–3.20
**In-hospital mortality at 14 days**	16	12	RR = 1.24, 95% CI 0.62–2.49
**In-hospital mortality at 28 days**	18	15	RR = 1.12, 95% CI 0.59–2.10

Abbreviations: NEWS2 = National Early Warning Score 2, NIV = non-invasive ventilation.

**Table 5:** Lactoferrin effectiveness in driving disease evolution towards primary endpoints according to age subgroup and gender.

**Age**	**Outcome**	**Lactoferrin**	**Placebo**	**Difference between Groups** **95% CI**	***p*-Value**	**RR** **95% CI**
**<65 years**	**Adverse outcome**	8/55	6/52	3% −10.38–16.13	0.6467	1.26 0.47–3.39
	**Positive outcome**	43/55	42/52	2.60% −12.86–17.72	0.7405	0.97 0.80–1.17
**≥65 years**	**Adverse outcome**	16/58	15/53	0.70% −15.61–17.22	0.9349	0.97 0.54–1.77
	**Positive outcome**	24/58	31/53	17.10% −1.44–34.07	0.0732	0.71 0.48–1.04
**Sex**	**Outcome**	**Lactoferrin**	**Placebo**	**Difference between groups** **95% CI**	***p*** **-value**	**RR** **95% CI**
**Females**	**Adverse outcome**	8/44	2/33	12.12 −3.96–26.58	0.1198	3.00 0.68–13.21
	**Positive outcome**	27/44	27/33	20.46 −0.26–38.03	0.0538	2.13 0.94–4.80
**Males**	**Adverse outcome**	16/69	19/72	3.20 −11.05–17.18	0.6613	0.88 0.49–1.57
	**Positive outcome**	40/69	46/72	5.92 −9.97–21.44	0.4728	1.16 0.77–1.76

**Table 6:** Adverse events in the two treatment arms.

	Lactoferrin	Placebo	Total	χ^2^	*p*-Value
**Significant bleeding events**	1	1	2	0.0027	0.9585
**Thoracic pain**	9	4	13	1.6680	0.1965
**Arrhythmias**	10	7	17	0.3590	0.5490
**ACS (STEMI/NSTEMI)**	1	1	2	0.0027	0.9585
**Heart failure**	3	3	6	0.0083	0.9275
**ALT elevation 3 × ULN**	13	13	26	0.0396	0.8422
**Pneumomediastinum**	2	2	4	0.0055	0.9410
**VTE and pulmonary thromboembolism**	3	3	6	0.0083	0.9275
**AKI**	3	5	8	0.6800	0.4094
**Bacterial infection including bacteremia**	9	6	15	0.4280	0.5129
**Diarrhea**	0	4	4	4.3650	0.0367
**Seizures**	1	0	1	0.9290	0.3351
**Rhabdomyolysis**	2	0	2	1.8670	0.1718
**Total**	57	49	106	0.3090	0.5782

Abbreviations: ACS = acute coronary syndrome, STEMI = ST-segment elevation myocardial infarction, NSTEMI = non-ST-segment elevation myocardial infarction, ALT = alanine aminotransferase, ULN = upper limit of normal (defined by local laboratory settings), VTE = venous thromboembolism, AKI = acute kidney injury.

## Data Availability

Data are available upon reasonable request to the corresponding author.

## Supplementary Materials

The following supporting information can be downloaded at https://www.mdpi.com/article/10.3390/nu15051285/s1, File S1: LAC trial study protocol.
