# Supplementary material for: Effect of Lactoferrin on Clinical Outcomes of Hospitalized Patients with COVID-19: The LAC Randomized Clinical Trial
Source: Nutrients. 2023 Mar 4;15(5):1285. doi: 10.3390/nu15051285 (PMC10005739; doi:10.3390/nu15051285)
Supplement: Supplementary file 1 [file nutrients-15-01285-s001.zip › nutrients-2237038-supplementary.pdf]

Supplement 1: LAC trial Protocol

LAC trial Protocol

Lactoferrin for treatment of Acute COVID-19 infection in hospitalized patients: a multicenter, double blind, randomized, placebo-controlled clinical

Version 1.6 (23/12/2020)

Index	Pag
**Contacts**	3
**Introduction**	4
**Rationale**	5
Hypothesis and clinical application	5
**Materials and Methods**	6
Study design	6
Objectives	6
Population	6
Intervention	7
Clinical and laboratory variables	9
Operative definitions	9
Safety	10
**Statistics**	11
Sample size calculation	11
Statistical analysis	11
Data management	11
**Ethics**	13
Informed consent, data management and ethical committee	13
**Financial agreements and insurance**	14
**Data ownership and results publication**	14
**Bibliography**	15

CONTACTS

Sponsor and principal investigator

Dr. Paolo Manzoni

S.C. Pediatria

Ospedale degli Infermi

Via dei Ponderanesi, 2

Ponderano, Biella

Email: paolomanzoni@hotmail.com

Scientific commitee and co-investigators

Dr. Paolo Manzoni°, Dr. Anna Lingua°, Dr. Aldo Tua°, Dr. Luca Grillenzoni°, Prof. Piera Valenti*, Prof. Pier Paolo Sainaghi§

°Ospedale degli Infermi

Via dei Ponderanesi, 2

Ponderano, Biella

*Università La Sapienza, Roma

§Università del Piemonte Orientale (UPO) and AOU "Maggiore della Carità", Novara

Statistics and data analysis

Dr. Paolo Manzoni, Dr. Danila Azzolina (UPO)

Study type

No profit

Results publication

Dr. Paolo Manzoni

INTRODUCTION

COVID-19 pandemic is rapidly spreading, despite the extraordinary measures adopted to contain it (Chang et al., 2020).

The causative agent of this pandemic is the novel Severe Acute Respiratory Syndrome Coronavirus 2 (SARS-CoV-2), an enveloped, single stranded RNA virus. The resulting disease, called COronaVIrus Disease 2019 (COVID-19), is characterized by a wide range of clinical manifestations, ranging from flu-like symptoms to gastrointestinal manifestations (Xiao et al., 2020; Lin et al., 2020), acute respiratory distress syndrome, disseminated intravascular coagulopathy (DIC), cardiac arrhythmias, stroke and even death (Avula et al., 2020; Kochi et al., 2020).

Coronaviruses (CoV) belong to the Coronaviridae family (Coronavirinae sub-family) and can infect a broad range of hosts. In humans, the clinical manifestations of CoV infection range from the common cold to a severe and even fatal disease, as already observed for the previous SARS and MERS outbreaks and now for COVID-19.

SARS-CoV-2 is one of the seven CoV family members able to infect humans and, even if it is genetically different, it belongs to the same CoV linage of the SARS causative agent.

Until 2019, there were 6 CoV known to be able to infect humans: HCoV-229E, HCoV-NL63, HCoV-OC43, HCoV-HKU1, SARS-CoV and MERS-CoV. Although SARS-CoV and MERS-CoV caused highly lethal outbreaks, the others were associated to upper airways mild infections (Chen et al., 2020; Dhama et al., 2019).

At the time of writing (15th November 2020), 54083176 SARS-CoV-2 infections occurred worldwide, resulting in 1313438 deaths. Globally, Italy is the 10th country for infections and the 6th for deaths (Dong et al., 2020).

Pharmacological therapies available to date do not specifically target the etiological agent of the disease and are mainly represented by antibiotics, heparin, steroids, anti-inflammatory drugs and supportive care.

Even if different antiviral agents and nutritional supplements have been proposed as potentially useful approaches to treat SARS-CoV-2, only few of them underwent clinical evaluation (Chang et al., 2020).

Lactoferrin (LF) is a 692 aminoacids single chain polypeptide, belonging to the transferrin family. This backbone forms two highly structured and homologue lobes (N and C lobe, respectively), each one able to bind a single iron ion (Fe^{+++}).

LF is mainly present in mammals' milk, but it could be detected also in other biological fluids (i.e. tears, saliva, mucus, etc) and in neutrophils' granules. In breast milk, its concentration is the highest in colostrum, while it gradually decreases in mature milk (Yang et al., 2018).

The commercially available bovine LF shows a very high structural homology to human LF and several studies in vitro, as well as in vivo, highlighted that both display similar activities.

Bovine LF is a non-toxic glycoprotein, globally marketed as GRAS (Generally Recognized As Safe) nutritional supplement.

LF activity has been evaluated in vitro against several viruses, including SARS (Severe Acute Respiratory Syndrome) causative coronavirus (Lang et al., 2011), which is strictly related to the new SARS-CoV-2. Furthermore, LF effects have been tested extensively in vitro (Campione et al., 2020; Mirabelli et al., 2020 BioRxiv; Campione et al., 2020 BioRxiv) and in vivo (Serrano et al., 2020; Campione et al, 2020 BioRxiv). LF has also be shown to positively modulate host responses to infections through its immunomodulatory and anti-inflammatory properties (Legrand et al., 2006).

Moreover, SARS-CoV-2 infection is known to be associated to a pro-inflammatory cytokine storm, where IL-6 plays a crucial role. Such hypercytokinemia results in inflammatory responses and iron homeostasis dysregulation, finally leading to cellular iron overload. Such iron accumulation inside the cells is known to favor both viral replication and reactive oxygen species formation, finally resulting in cellular damage and organ dysfunction (Rosa et al., 2017).

LF ability to inhibit viral entry into host cells mainly relies on different mechanisms: 1) high affinity binding to cell surface molecules such as heparan sulphate proteoglycans (HSPG); 2) direct binding to viral particles through the interaction with the spike protein; 3) a combination of both previously described mechanisms (Campione et al., 2020 BioRxiv).

Several studies highlighted that viral entry into the host cell is a very complex process (Spear, 2004; Sapp and Bienkowska-Haba, 2009; Berlutti et al., 2011). Moreover, recent studies highlighted that SARS-CoV-2 entry involves cell surface molecules, including ACE2 receptor (Wang et al. 2020).

HSPG have been identified as the initial point of adhesion on cell surface: moreover, here their concentration could be increased to favor a more specific entry receptor binding (Andersen et al., 2004).

SARS-CoV sticks to host's cells through HSPG binding (Belting et al., 2003) and HSPG are also the LF binding site on host cell surface (Carvalho et al., 2004).

In a 2011 study using SARS pseudo-coronavirus (SARS-CoV), Lang and coworkers identified ACE2 as one of the SARS-CoV functional receptors, which is responsible for spike protein binding and for the following SARS-CoV entry into the host cells. ACE2 is highly expressed on human pulmonary alveolar epithelial cells, on small intestine enterocytes and on kidney proximal tubular cells (Lang et al., 2011).

RATIONALE

In 2011 Lang and coworkers demonstrated that LF was useful in treating SARS infection. Later, other in vitro studies added new interesting evidences to such results.

LF gene expression is known to be increased in SARS patients, where LF serum levels and its release by neutrophils are increased (Serrano et al., 2020).

LF shows a potent anti-viral action against both naked and enveloped RNA or DNA viruses (Berlutti et al., 2011; Wakabayaski et al., 2014 and references included in both review) (see annex 1). This anti-viral action is mostly related to its competitive binding to HSPG and/or viral surface components (Valenti and Antonini, 2005).

LF has been demonstrated to be able to prevent SARS-CoV-2 internalization in vitro, through a mechanism similar to that described above, namely HSPG and spike binding (Campione et al., 2020 BioRxiv).

Such evidences were confirmed also in vivo on a limited number of Spanish and Italian patients (Serrano et al., 2020; Campione et al., 2020 BioRxiv).

Taken together, these evidences suggest that LF could be a useful therapeutic option in reducing COVID-19 severity (Mirabelli et al., 2020).

HYPOTHESIS AND CLINICAL APPLICATION

Our hypothesis is that, in hospitalized COVID-19 patients, a daily administration of bovine LF in addition to standard therapy for 1 month could be useful in limiting disease severity and progression, as well as in reducing swab time to negativization and morbidity.

To the best of our knowledge, in such patients, no prospective, placebo-controlled studies have evaluated oral LF efficacy in improving clinical recovery and disease resolution.

MATERIALS AND METHODS

STUDY DESIGN

No profit, multicenter, double blind, randomized with nutritional supplement (bovine lactoferrin – Mosiac 200 mg) study.

OBJECTIVES

The main study objective is the evaluation of lactoferrin (LF) efficacy, compared to placebo, in limiting COVID-19 progression and severity in hospitalized patients, when orally administered (BLF-Mosiac) in addition to standard of care therapy.

PRIMARY ENDPOINT

The primary endpoint will be the evaluation of oral bovine LF (BLF-Mosiac) efficacy compared to placebo in modifying at least one of the following: 1) reduction of a composite event rate consisting of one of two events, whichever occurred first: hospitalizations in intensive care units (ICU) (due to any cause) or in-hospital death; 2) increase of the proportion of a composite event rate consisting of one of two events, whichever occurred first: discharge from hospital within 14 days from enrollment or NEWS2 ≤ 2 for at least 24 hours within 14 days from enrollment.

SECONDARY ENDPOINTS

The secondary endpoints will be defined as the evaluation of a possible superiority of the bovine LF intervention compared to the standard treatment in:

1. variation (improvement or worsening) of NEWS2 score, from the day of hospitalization to that measured at 7, 14 and 21 days;
2. difference in oxygen supplementation need and its duration;
3. need of non-mechanical assisted ventilation (including HFNC – high flow nasal cannula and properly defined non-invasive ventilation (NIV) considering either continuous positive airway pressure (CPAP) or bilevel positive airway pressure (BiPAP) through a noninvasive interface, such as a face or nasal masks);
4. need of mechanical ventilation;
5. variation of in-hospital mortality rate at 14 and 28 days from enrollment;
6. variation in C-reactive protein (CRP), IL-6 and ferritin plasma levels during hospitalization;
7. adverse events related to the treatments;
8. Variation of D-dimer and hepcidin serum levels;
9. Swab time to negativization;
10. C reactive protein levels < 1 mg/ml within 10 days and time needed to reach it;
11. $pO_2/FiO_2 > 350$ in two consecutive evaluations, only for the subgroup of patients with a ratio < 300 at the time of hospitalization and in the presence of radiologically and clinically documented pneumonia.

POPULATION

All COVID-19 patients admitted to non-ICU wards of the participating centers, with or without respiratory failure, will be screened for eligibility.

Patients recruitment will involve two centers: Ospedale degli Infermi (Ponderano, Biella) and AUO Maggiore della Carità (Novara).

In Biella, patients will be recruited in the COVID dedicated wards: i.e. medicine, pneumology, physiatry, etc, while intensive care units will not be included.

In Novara, all COVID dedicated wards will be involved: COVID-Neurology, COVID-Medicine 1, COVID-pneumology, COVID-Surgery, COVID-Cardiosurgery.

INCLUSION CRITERIA

1. Hospitalization in non-ICU COVID-19 dedicated wards, with virological confirmation of SARS-CoV-2 infection either by reverse-transcription polymerase chain reaction or by third generation antigenic test (according to local guidelines) from nasopharyngeal swabs samples;
2. Age ≥ 18 years;
3. Onset of COVID-19 symptoms within 12 days.

EXCLUSION CRITERIA

1. Denial of the informed consent;
2. Need for immediate ICU admittance;
3. Advanced cancer history;
4. Known allergies or intolerances to lactoferrin;
5. Being already in treatment with lactoferrin at hospital admission;
6. End stage renal disease (stage V, GFR<15ml/min);
7. Critical clinical conditions suggestive of imminent death;
8. Inability to tolerate or clinical conditions that might contraindicate oral treatments.

<u>INTERVENTION</u>

Enrolled patients will be randomized to two groups:

- Group A – standard therapy for COVID-19 + oral bovine lactoferrin 400 mg (2 capsules of Mosiac 200) every 12 h (i.e. fixed dose of 800 mg/day) for 30 days, between meals;
- Group B (control) – standard therapy for COVID-19 + placebo (identical capsules containing the same quantity of an inert component, corn starch powder), following the same posologic scheme.

<u>It has to be pointed out that both intervention and placebo will be administered in addition to the standard of care pharmacological treatments actually used in clinical practice for COVID-19 management</u>.

All drugs used to treat COVID-19 patients will be prescribed according to their marketing recommendations and to the state of the art, as well as according to the clinical presentation of each patient. Moreover, pharmacological treatments will be administered in clinical practice, according to the most updated protocols and scientific evidences.

Lactoferrin purity, activity and integrity will be certified by an external laboratory, issuing the relative certification of quality (Prof. Valenti, Università La Sapienza, Roma).

Both participating centers will use an ex-novo Mosiac batch, manufactured ad hoc for the study.

Pharmaguida srl will provide both intervention and placebo, free of charge, and will cover the costs for quality control evaluations.

ACTIVITY FLOW CHART

	Screening and treatment start (within 24 h from hospitalization)	Every day during treatment (1 month)	Every 7 days during hospitalization	Discharge	From day 7 to day 18 since discharge
Informed consent	X				
Assessment of inclusion/exclusion criteria	X				
Randomization	X				
Clinical score	X	X		X	X
Clinical and pharmacological history	X				
Adverse events recording		X		X	X
Need of O_2 recording	X	X		X	X
Arterial blood gas analysis with PaO_2/FiO_2 determination	X		X	X	X
Molecular swab	X		X	X	X
Serum ferritin, IL-6, D-dimer levels determination	X		X	X	
Serum hepcidin determination	X			X	

PATIENTS FLOW, SCREENING AND RANDOMIZATION

Patients admitted to the Emergency Departments of the participating centers will be screened for eligibility by the clinical staff once admitted to COVID-19 dedicated wards.

Whether eligibility criteria were satisfied, patients will be asked to sign and date a specific informed consent form after a detailed explanation of the document. Eligible patients from both centers will be registered in a centralized database hosted by Università del Piemonte Orientale (UPO)/Azienda Ospedaliero-Universitaria “Maggiore della Carità” (Novara) IT department. Pseudo-anonymized patients' data will be recorded by each clinical center staff in a web-based dedicated CRF (case report form).

Patients signing the informed consent will be randomized and allocated to the LF or placebo arm based on previously prepared randomization lists.

In each clinical center, randomization procedure will be carried out in a competitive and balanced manner. Randomization will be performed with a procedure based on permuted block scheme with a block size of 4 (Rosemberg et al., 2002), with an allocation ratio of 1:1 to assure a balanced distribution in each study arm. Each block will consist of a specific number of allocations, randomly sorted (Rosemberg et al., 2002).

The randomization list will be managed through the REDCap randomization module.

The hospital pharmacy of each participating center will not be involved in patients recruitment, but it will prepare sequentially numbered opaque-sealed envelopes, containing the allocation code, and will store the pairing list in a place not accessible to the recruiting investigators.

The sealed envelopes will be sequentially opened at the time of randomization, after obtaining the informed consent.

Blindness will be preserved by the separation of the pharmacy staff (who will prepare the randomization lists and will manage the delivery of the study products (of identical appearance)) from the investigator staff. The two staffs will be separated also from a logistic point of view, being located in different places inside the hospital structures.

Screening and randomization procedures, as well as intervention administration, will be performed within 24 hours from ward admission.

It has to be pointed out that all enrolled patients will start the standard of care therapy independently from randomization and regardless of treatment arm, as the basal therapeutic regimen will be identical for both groups.

CLINICAL AND LABORATORY VARIABLES

For each enrolled patient we will collect clinical information and laboratory findings that are routinely employed in clinical practice and easily achievable from medical records:

- COVID-19 diagnostic information (date of the diagnosis, symptoms onset date);
- Symptoms at disease onset (cough, dyspnea, fever, anosmia, dysgeusia, musculoskeletal pain, gastrointestinal disorders, etc);
- Ongoing COVID-19 treatment;
- Existing comorbidities;
- Medical history and related ongoing therapy (if applicable);
- Vital parameters (at enrollment, daily during hospital stay, at the time of discharge);
- COVID-19 clinical severity score: we will use the NEWS score (see annex 2), that will be recorded twice a day for the whole hospital stay;
- Laboratory parameters (C-reactive protein (CRP), lactic dehydrogenase (LDH), erythrocyte sedimentation rate (ESR), lymphocyte count, D-dimer, serum albumin, hemoglobin, cardiac troponin, creatinine, alanine transaminase (ALT), aspartate aminotransferase (AST), bilirubin, ferritin, IL-6, hepcidin);
- Daily PaO_2/FiO_2 (this is an index of alveolar respiration: in an healthy patient it is 450 (ratio between 95 (the partial arterial oxygen pressure) and 21 (the percentage of inspired air oxygen)); if it is higher than 350 it would be considered as “normal” , while values lower than 200 are associated to a severe respiratory failure);
- In-hospital therapies (starting date, type of therapy);
- Need of oxygen supplementation;
- Days of oxygen supplementation;
- ICU transfer and its duration;
- Fulfillment of discharging criteria;
- Need of mechanical ventilation;
- Days of mechanical ventilation;
- Concomitant diagnosis of bacterial pneumonia.

OPERATIVE DEFINITIONS

- SARS-CoV-2 related hospitalization: any hospital admission of patients > 18 years (for any cause directly or indirectly related to COVID-19)
- Non-invasive ventilation: any ventilation approach more invasive than oxygen supply through conventional mask (including approaches not requiring intubation: high flow nasal cannulas (HFNC), nasal continuous positive airway pressure (nCPAP), synchronized intermittent mandatory ventilation (SIMV), synchronous positive pressure ventilation (SIPP), high frequency oscillatory ventilation (HFOV), extracorporeal membrane oxygenation (ECMO), etc)
- Clinical severity score: we will use the NEWS2 score (see annex 2). This score will be calculated twice a day (or according to clinical needs) and will be recorded on CRF
- Discharge: condition according to which a patient did not need oxygen supplementation or venous accesses, shows stable vital parameters when breathing ambient air since 72 hours, and did not show COVID-19 related complications affecting its general conditions stability.

SAFETY

Bovine LF is a nutraceutical supplement with notification of GRAS (Generally Recognized As Safe) officially attributed by the US Food and Drug Administration (FDA), without any evidence of intolerances or safety issues. No adverse reactions are expected following its administration. As LF is a glycoprotein present in both human and bovine milk (and not a whey protein such as casein or lactoalbumin) no allergic reactions have been described to date.

According to the present protocol, we will perform vital parameters monitoring for the whole study duration and we will perform a systematic detection and recording of grade 3 and 4 adverse events (AE), as well as of severe adverse events (SAE).

The need of treatment withdrawal, along with laboratory findings during the time of the study will be reported in medical records.

STATISTICS

SAMPLE SIZE CALCULATION

The trial is powered for the primary endpoint and sample size is determined using a model built on historical data for the same endpoint.

Pre-trial data from the two recruiting centers estimated a 25% ICU admission rate, a 30% non-invasive ventilation need, a 15% in-hospital mortality, and an average hospital stay of 16 days.

Sample size needed for the study has been determined considering a two-sided t-test for two independent samples according to the following two possible scenarios:

- Scenario 1:
  - Alpha level of 0.05;
  - Overall power of 0.8;
  - Cohen effect of 0.44 (corresponding to a small/medium standardized effect (Cohen, 1977) of a 14 day discharge rate of 60% for controls and of 80% for treated patients).
- Scenario 2:
  - Alpha level of 0.025, corrected according to Bonferroni method (overall alpha level = 0.025*2 = 0.05);
  - Overall power of 0.8;
  - Cohen effect of 0.44 (corresponding to a small/medium standardized effect (Cohen, 1977) of a 14 day discharge rate of 60% for controls and of 80% for treated patients).

The sample size needed for the study resulted in 80 patients/arm according to the first scenario and in 97 patients/arm according to the second scenario.

Sample size calculations were performed using the R 3.6.1 software (R Core Team, 2015) and the pwr package (Champely, 2018).

STATISTICAL ANALYSIS

Continuous variables will be expressed in terms of median and interquartile range (IQR) (defined as measure of central tendency and dispersion), while categorical variables will be presented as percentage (absolute numbers).

Primary endpoint will be calculated on an intention-to-treat population basis, by performing a bilateral t-test to evaluate differences between proportions and to calculate the relative risk (RR) with a 95% confidence interval (95% CI).

Secondary endpoints will be evaluated by performing a bilateral t-test to evaluate differences between proportions for binary endpoints (Pearson χ^2 test) and by performing the Mann-Whitney U test to evaluate differences between median values for continuous endpoints.

Differences between event timing will be evaluated through a Long Rank test and the event free survival will be evaluated through the Kaplan-Meier method.

In all statistical analyses significance threshold will be set at 0.05.

DATA MANAGEMENT

The global objective of the data management process is to guarantee a high quality data delivery to satisfy good clinical practice (GCP) requirements as well as to support statistical analysis and reporting needs. All phases of the data collection process are involved, i.e. data acquisition, elaboration and usage.

Clinical data will be collected in a longitudinal database to facilitate research procedures.

Patients data will be recorded on a web-based support (REDCap 8.10.18) with restricted access.

REDCap is a software developed to manage data collection in the research field.

The EDC (electronic data capture) will be developed for:

- Database design, building and implementation;
- Data quality managing allowing inserted data checking (format, interval, etc);
- Manual or automatic study monitoring query creation;
- Data harmonization;
- Data export in formats suitable for statistical analysis.

During this study, data will be collected using investigator-defined forms, to facilitate data collection and to allow an immediate availability of the anonymous data.

Data collection and consultation will be protected through the definition of user-sensitive passwords.

Patients data will be recorded in an anonymized form and will be stored in a protected and secured place.

The data management system is validated according to FDA 21 CFR part 11. Encrypted data transmission will occur through the SSL (secure socket layer) technology and all changes to the stored data will be recorded by adding a digital timestamp.

Study database will be saved on a firewall protected server, located in a secured place.

To assure clinical data security and integrity, at least one person on each center will be in charge of data monitoring according to ICH-GCP (E6) guidelines and the approved standard operative procedures.

The study will be conducted according to the Declaration of Helsinki and to all the local and international regulations, i.e. the personal data protection act.

A written informed consent will be obtained from all enrolled patients before data collection.

ETHICS

INFORMED CONSENT, DATA MANAGEMENT AND ETHICAL COMMITTEE

This study will be conducted in accordance to the present protocol and to the good clinical practice procedures (according to DM 15/07/1997 n. 162 and to DM 24/06/2003 n. 211 and the following modifications).

Study protocol and all the related documents will be evaluated by the ethical committee of the Ospedale Maggiore della Carità, Novara.

From an ethical point of view, it has to be noted that data will be collected from hospitalized patients' medical records or from outpatients' records.

Investigators will inform patients about the study and will ask for their consent to collect data from their medical records. Investigators will also document both patient information and informed consent acquisition procedures.

For study reports and publications data will be used only disaggregated and anonymously.

All collected data (in ad hoc forms) will be stored anonymously in an electronic database.

FINANCIAL AGREEMENTS AND INSURANCE

As the study does not involve the use of experimental drugs, the enrolled patients will not incur in additional risks.

As the study does not involve procedures or treatments different from the current clinical practice, a study-dedicated insurance is not mandatory and there will not be additional costs for the national health system.

This is a no profit study, so ethical committee fees are not expected.

Intervention and placebo will be provided, free of charge, by Pharmaguida srl.

No commercial companies will be involved neither in study conceptualization, design and registration, nor in data collection, data analysis, results interpretation and publication decisions.

DATA OWNERSHIP AND RESULTS PUBLICATION

The promoting center will maintain the full property of data.

According to DM 17/12/2004, study sponsor will assure results dissemination through congresses participation and scientific publications.

Study sponsor will send a study report to the competent authorities within 12 months from study closure.

BIBLIOGRAPHY

1. Andersen JH et al. Anti-HSV activity of lactoferrin and lactoferricin is dependent on the presence of heparan sulphate at the cell surface. J Med Virol 2004; 74: 262-71
2. Avula A et al., COVID-19 presenting as stroke. Brain Behav Immun 2020; doi: 10.1002/jmv.20171
3. Belting M. Heparan sulfate proteoglycan as a plasma membrane carrier. Trends Biochem Sci 2003; 28: 145-151
4. Berlutti F et al. Antiviral properties of lactoferrin – A natural immunity molecule. Molecules 2021; 16: 6992-7018. Doi: 10.3390/molecules16086992
5. Campione E et al. Lactoferrin as protective natural barrier of respiratory and intestinal mucosa against coronavirus infection and inflammation. Int J Mol Sci 2020; 21(14): 4903; doi: 10.3390/ijmn21144903
6. Campione E et al. Pleiotropic effect of lactoferrin in the prevention and treatment of COIVD-19 infection: in vivo, in silico and in vitro preliminary evidences. BioRxiv 2020, in print
7. Carvalho CAM et al. Inhibition of Mayaro virus infection by bovine lactoferrin. Virology 2014; 452-458: 297-302
8. Chang R et al. Lactoferrin as potential preventative and adjunct treatment for COVID-19. Int J Antimicrob Agents 2020 Sep; 56(3): 106118. doi: 10.1016/ijantimicag.2020.106118
9. Chen Y et al. Emerging coronaviruses: genome structure, replication and pathogenesis. J Med Virol 2020; 92: 418-23
10. Dhama K et al. Coronavirus disease 2019 – COVID-19. Clin Microbiol Rev 2020; Jun 24; 33(4):e00028-20 doui 10.1128/CMR.00028-20
11. Dong E et al. An interactive web-based dashboard to track COIVD-19 in real time. Lancet Infect Dis 2020; 20: 533-534 https://doi.org/10.1016/S1473-3099(20)30120-1
12. Kochi AN et al. Cardiac and arrhythmic complications in patients with COVID-19. Journal of Cardiovascular Electrophysiology (2020) diu: 10.1111/jce.14479
13. Lang J et al. Inhibition of SARS pseudovirus cell entry by lactoferrin binding to heparan sulfate proteoglycans. PLoS One 2011; 6: e23710 doi: 10.1371/journal.pone.0023710
14. Legand D et al. Interactions of lactoferrin with cells involved in immune function. Biochem Cell Biol 2006; 84: 282-90 doi: 10.1139/o06-045
15. Lin L et al. Gastrointestinal symptoms of 95 cases with SARS-CoV-2 infection. Gut (2020) doi: 10.1136/gutjnl-2020-321013
16. Mirabelli C et al. Morphological cell profiling of SARS-CoV-2 infection identifies drug repurposing candidates for COVID-19. BioRxiv 2020, in print
17. Rosa L et al. Lactoferrin: a natural glycoprotein involved in iron and inflammatory homeostasis. Int J Mol Sci 2017; 18(9): 1985 doi: 10.3390/ijms18091985
18. Sapp M Bienkowska-Haba M. Viral entry mechanisms: human papillomavirus and a long journey from extracellular matrix to the nucleus. FEBS J 2009; 276: 7206-16
19. Serrano G et al. Liposomal lactoferrin as potential preventative and cure for COVID-19. Int J Res Health Sci 2020; 8(1): 8-15
20. Spear PG. Herpes simplex virus: receptors and ligands for cell entry. Cell Microbiol. 2004; 6: 401-10 doi: 10.1111/j.1462-5822.2004.00389.x
21. Valenti P, Antonini G. Lactoferrin: an important host defence against microbial and viral attack. Cell Mol Life Sci 2005 Nov; 62(22): 2576-87 doi: 10.1007/s00018-005-5372-0
22. Wakabayaski H et al. Lactoferrin for prevention of common viral infections. J Infect Cheother 2014; 20: 666-671
23. Wang Q, et al. Structural and functional basis of SARS-CoV-2 entry by using human ACE2. Cell 2020 May 14; 181(4): 894-904.e9
24. Wang Y et al. Lactoferrin for the treatment of Covid-19. Experimental and Ther Medicine 2020; 20: 272
25. Xiao F et al. Evidence for gastrointestinal infection of SARS-CoV-2. Gastroenterology (2020) doi: 10.1053/j.gastro.2020.02.055
26. Yang Z et al. Concentration of lactoferrin in human milk and its variation during lactation in Chinese populations. Nutrients 2018; 10(9): 1235 doi: 10.3390/nu10091235
27. Rosemberger WF, Lachin JM. Randomization and the clinical trial; Wiley Online Library 2002, ISBN 0-471-72210-3
28. Cohen J. Statistical power analysis for the behavioral sciences (revised ed) 1977

29. Core Team R: A language and environment for statistical computing; R Foundation for statistical computing: Vienna, Austria, 2015
30. Champely D Ekstrom C, Dalgaard P, Gill J, Weibelzahl S, Anandkumar A, Ford C, Volcic R, De Rosario H, De Rosario MH. Package "pwr" R package version 2018, 1-2

ANNEX 1

Table I. The antivirus activities of LF for some viruses.

Authors, year	Type of virus	Enveloped/naked	DNA/RNA	Sources of LF	Mechanism	(Refs.)
Oda *et al*, 2020	Influenza A	Enveloped	RNA	Bovine	Interfering with the fusogenic function of viral hemagglutinin	(28)
Sano *et al*, 2003	RSV	Enveloped	RNA	Human	Modulating RSV-induced IL-8 secretion and binding to RSV F protein	(29)
Pietrantoni *et al*, 2003	Adenovirus	Naked	DNA	Bovine	Binding to the adenovirus penton base and competing with viral particles for cell membrane HS inserted in target cell membranes	(30)
Lang *et al*, 2011	SARS-CoV	Enveloped	RNA	Bovine	Enhancing Natural killer cell activity and stimulating neutrophil aggregation and adhesion, binding to the heparan sulfate glycosaminoglycan (HSPG) and blocking the preliminary interaction between SARS-CoV and host cells	(31)
Chen *et al*, 2017	Dengue Virus	Enveloped	RNA	Bovine	Interacting with Heparan Sulfate, Low-Density Lipoprotein Receptor and DC-SIGN	(32)
Weng *et al*, 2005	Enterovirus 71	Naked	RNA	Bovine	Binding to viral protein 1 protein and host cells	(33)
Pietrantoni *et al*, 2015	Toscana Virus	Enveloped	RNA	Bovine	Binding to Heparan Sulphate	(34)
Beljaars *et al*, 2004	CMV	Enveloped	DNA	Human	Inhibition of CMV cell entry and indirect activities of lactoferrin on CMV infections via stimulation of the immune system	(35)
Ammendolia *et al*, 2007	Herpes Simplex Virus type 1 (HSV-1)	Enveloped	DNA	Bovine	Competing with HSV-1 for heparan sulphate receptor on cell surface and affecting a post-entry step of viral infection by preventing VP-16 from being translocated to the nucleus	(36)
Ishikawa *et al*, 2013	MNV	Naked	RNA	Bovine	Inducing the expression of anti-viral cytokine mRNA, such as IFN-a and IFN-b, which are involved in the inhibition of MNV replication in the early phase of infection	(37)

RSV, Respiratory syncytial virus; IL, interleukin; HS, heparan sulfate; SARS-CoV, severe acute respiratory syndrome coronavirus; HSPG, heparan sulfate proteoglycans; DC-SIGN, dendritic cell-specific intercellular adhesion molecule 3-grabbing non-integrin; CMV, cytomegalovirus; MNV, mouse norovirus; IFN, interferon.

(From: Wang Y et al. Exp Ther Med 2020)

ANNEX 2

NEWS score results from the sum of the single scores, and corresponds to the clinical conditions severity index:

0 = to be monitored every 12 hours

1-4 = to be monitored minimum every 4-6 hours

≥5 or 3 in a single parameter = to be monitored every hour

≥7 = need of continuous monitoring

N.E.W.S.2

National Early Warning Score

PHYSIOLOGICAL PARAMETERS	3	2	1	0	1	2	3
Respiratory rate/min	≤8		9-11	12-20		21-24	≥25
Oxygen saturation %	≤91%	92-93%	94-95%	≥96%			
Supplementary oxygen		YES		NO			
Temperature °C	≤35.0°		35.1° - 36.0°	36.1° - 38.0°	38.1° - 39.0°	≥39.1°	
Systolic pressure mmHg	≤90	91-100	101-110	111-219			≥220
Heart rate/min	≤40		41-50	51-90	91-110	111-130	≥131
Consciousness				Alert			Verbal call, coma

(From:

Royal College of Physicians. National Early Warning Score (NEWS): standardizing the assessment of acute-illness severity in the NHS. 2012. Available from: https://www.rcplondon.ac.uk/projects/outputs/national-early-warning-score-news-2

Royal College of Physicians. NEWS2: additional implementation guidance. 2020. Available from: https://www.rcplondon.ac.uk/projects/outputs/news2-additional-implementation-guidance

Gidari A, De Socio GV, Sabbatini S & Francisci D. (2020) Predictive value of National Early Warning Score 2 (NEWS2) for intensive care unit admission in patients with SARS-CoV-2 infection. Infectious Diseases 52(10) https://doi.org/10.1080/23744235.2020.1784457)
